# Association between Inflammatory Bowel Disease and Cholelithiasis: A Nationwide Population-Based Cohort Study

**DOI:** 10.3390/ijerph15030513

**Published:** 2018-03-14

**Authors:** Chien-Hua Chen, Cheng-Li Lin, Chia-Hung Kao

**Affiliations:** 1 Digestive Disease Center, Show-Chwan Memorial Hospital, Changhua 500, Taiwan; showchench@yahoo.com.tw; 2 Department of Food Science and Technology, Hungkuang University, Taichung 433, Taiwan; 3 Chung Chou University of Science and Technology, Yuanlin Township, Changhua County 510, Taiwan; 4 Management Office for Health Data, China Medical University Hospital, Taichung 404, Taiwan; orangechengli@gmail.com; 5 College of Medicine, China Medical University, Taichung 404, Taiwan; 6 Graduate Institute of Clinical Medical Science, School of Medicine, College of Medicine, China Medical University, No. 2, Yuh-Der Road, Taichung 404, Taiwan; 7 Department of Nuclear Medicine and PET Center, China Medical University Hospital, Taichung 404, Taiwan; 8 Department of Bioinformatics and Medical Engineering, Asia University, Taichung 413, Taiwan

**Keywords:** cholelithiasis, inflammatory bowel disease, Crohn’s disease, ulcerative colitis

## Abstract

We assessed the subsequent risk of cholelithiasis development in patients with inflammatory bowel diseases (IBDs) such as Crohn’s disease (CD) or ulcerative colitis (UC). We identified 8186 patients who aged ≥20 years and were diagnosed with IBD between 2000 and 2010 as the study cohort. A total of 8186 patients without IBD were selected by frequency-matching according to age, sex, comorbidities, and the index date of diagnosis, and they were identified as the control cohort. To measure the incidence of cholelithiasis, all patients were followed up until the end of 2011. The risk of developing cholelithiasis, either gallbladder stone disease (GSD; adjusted hazard ratio (aHR) = 1.76, 95% CI = 1.34–2.61) or common bile duct (CBD) stones and intrahepatic stones (IHSs; aHR = 2.78, 95% CI = 1.18–6.51), was higher for the CD cohort than for the non-IBD cohort after adjusting for age, sex, and comorbidities of hyperlipidemia, diabetes, liver cirrhosis, hypertension, chronic obstructive pulmonary disease, stroke, coronary artery disease, and hepatitis C virus infection. However, UC was related to the development of GSD (aHR = 1.44, 95% CI = 1.19–1.75) but not to CBD stones and IHSs (aHR = 1.70, 95% CI = 0.99–2.91). Our population-based cohort study demonstrated that CD is related to the development of cholelithiasis, including GSD alone and non-GSD-associated cholelithiasis. However, UC is only related to the development of GSD alone.

## 1. Introduction

Cholelithiasis consists of gallbladder stone disease (GSD), intrahepatic stones (IHSs), and common bile duct (CBD) stones, which are distinguished based on the stone distribution in the biliary trees. Cholelithiasis is one of the most commonly encountered medical conditions in gastrointestinal departments, and it may lead to cholecystitis, cholangitis, pancreatitis, and biliary tract cancer. GSD is the major type of cholelithiasis, and the prevalence of cholelithiasis in Taiwan has been reported to be approximately 5–10% [1,2,3]. Cholelithiasis is considered to be the migration of a stone from the gallbladder that may result in concomitant gallbladder and CBD stones, and it affects approximately 10–15% of patients with CBD [4]. However, approximately 85–90% of patients with CBD stones alone are considered to have de novo stones in the CBD. The development of IHSs or CBD stones is mainly related to the anatomical stricture or dilatation of the biliary trees caused by inflammation. Moreover, the stones may dislodge from the gallbladder or the intrahepatic ducts into the CBD. The development of GSD is reportedly related to age, female sex, and metabolic disorders.

Inflammatory bowel disease (IBD) includes Crohn’s disease (CD) and ulcerative colitis (UC). With enhanced disease awareness, improved diagnostic tools, and an increasingly westernized lifestyle, the incidence of IBD has rapidly increased in Asia [5]. Extraintestinal manifestations are common in patients with IBD, but most clinical studies have only focused on the viral hepatitis and hepatotoxicity caused by IBD-related drugs [6]. However, several laboratory studies have proposed that IBD, particularly CD, can predispose to the development of GSD by impairing the enterohepatic reabsorption of bile acids and the gallbladder emptying [7,8,9]. Furthermore, the IBD-related drugs can lead to GSD by inducing hemolysis [10]. Whereas primary sclerosing cholangitis, relatively common in UC, can predispose to the stone formation in the intrahepatic and extrahepatic biliary trees [11].

CD has been shown to be reportedly related to the development of GSD in epidemiological studies. However, associations between CD and IHSs or CBD stones remain unknown [12,13]. Moreover, the association between UC and cholelithiasis remains controversial [14,15]. Overall, the association between the subtypes of IBD and the subtypes of cholelithiasis remains debated in epidemiological studies. In this nationwide population-based cohort study, we hypothesized that a history of IBD might be related to the subsequent development of cholelithiasis. We analyzed data from the Longitudinal Health Insurance Research Database 2000 (LHID2000) of Taiwan to investigate the association of CD or UC with the subsequent development of cholelithiasis, including GSD alone and non-GSD-associated cholelithiasis.

## 2. Methods

### 2.1. Data Source

The National Health Insurance (NHI) program of Taiwan was established in 1995 and provides comprehensive and universal health care coverage to approximately 99% of the 23.74 million residents of Taiwan [16]. This study was conducted using the LHID2000, which comprises the data of 1,000,000 randomly sampled beneficiaries of the NHI program (nearly 5% of the residents of Taiwan). The details of this insurance program and the LHID2000 are described in previous studies [17,18]. In the LHID2000, diseases are diagnosed according to the codes in the 2001 edition of the International Classification of Diseases, Ninth Revision, Clinical Modification (ICD-9-CM). The study was approved by the Ethics Review Board of China Medical University and Hospital (approval no. CMUH104-REC2-115-CR2); the need for informed consent was waived by the ethics review board because the data obtained from the LHID2000 had been de-identified.

### 2.2. Sampled Participants

The two cohorts in this retrospective cohort study were IBD and non-IBD cohorts. For the IBD cohort, we identified patients aged ≥20 years who were newly diagnosed with IBD (ICD-9-CM codes 555–556), including UC (ICD-9-CM code 556) and CD (ICD-9-CM code 555), from 1 January 2000 to 31 December 2010, and who had complete age and sex information. The index date for the IBD cohort was set as the first IBD diagnosis date. Patients with a history of cholelithiasis (ICD-9-CM code 574) before the index date were excluded. The non-IBD cohort included individuals without IBD and without a history of cholelithiasis before recruitment. For each IBD patient, one non-IBD individual was selected and frequency-matched according to age (five-year range), sex, and baseline comorbidities of hyperlipidemia (ICD-9-CM code 272), diabetes (ICD-9-CM code 250), liver cirrhosis (ICD-9-CM codes 571.2, 571.5, and 571.6), alcohol-related illness (ICD-9-CM codes 291, 303, 305, 571.0, 571.1, 571.2, 571.3, 790.3, A215, and V11.3), hypertension (ICD-9-CM codes 401–405), chronic obstructive pulmonary disease (COPD; ICD-9-CM codes 491, 492, and 496), obesity (ICD-9-CM code 278), stroke (ICD-9-CM codes 430–438), coronary artery disease (CAD; ICD-9-CM codes 410–414), hepatitis B virus infection (ICD-9-CM codes V02.61, 070.20, 070.22, 070.30, and 070.32), and hepatitis C virus (HCV) infection (ICD-9-CM codes 070.41, 070.44, 070.51, 070.54, 070.70, 070.71, and V02.62). The index date assigned to the non-IBD individual was the same date as that of the corresponding patient with IBD. All included patients were followed up until cholelithiasis diagnosis, withdrawal from the NHI program, death, or 31 December 2011. Emigration from Taiwan and death were the major reasons for withdrawal from the NHI program, and the analysis included both cause-specific and non-cause-specific deaths when the causes were identified and registered in the National Health Insurance Research Database (NHIRD) of Taiwan.

### 2.3. Statistical Analysis

The distribution of age, sex, and comorbidities was compared between the IBD and non-IBD cohorts by using the chi-squared test for categorical variables and the Student’s *t*-test for continuous variables. To assess the difference in the cumulative incidence rates of cholelithiasis between the IBD and non-IBD cohorts, Kaplan-Meier analysis and the log-rank test were applied. The incidence density rates of cholelithiasis were estimated by dividing the number of cholelithiasis cases by the number of person-years for each risk factor, and the rates were then stratified by age group, sex, and comorbidity. Univariable and multivariable Cox proportional hazard regression models were employed to examine the effect of IBD on the risk of developing cholelithiasis. The results are expressed as hazard ratios (HRs) with 95% confidence intervals (CIs). The multivariable-adjusted models included all of the statistically significant covariates that were identified in the univariable model. Further analysis was performed to determine whether patients with IBD exhibit significant risks of developing cholelithiasis, including GSD and non-GSD, or various types of IBD, including UC and CD. All statistical analyses were performed using the SAS 9.3 software package for Windows (SAS Institute, Cary, NC, USA). The level of significance level was set at *p* < 0.05, and the tests were two-tailed.

## 3. Results

We identified 8186 patients with IBD and matched them with 8186 individuals without IBD according to age, sex, and comorbidities (Table 1). The majority of patients in the IBD cohort were aged ≤49 years (58.9%), and the prevalence of IBD decreased with age. The mean age was 47.7 ± 16.8 years in the IBD cohort and 47.9 ± 17.2 in the non-IBD cohort. In these cohorts, the most prevalent comorbidity was hypertension (27.9%), followed by hyperlipidemia (17.7%), CAD (15.4%), and COPD (11.3%).

**Figure 1:**
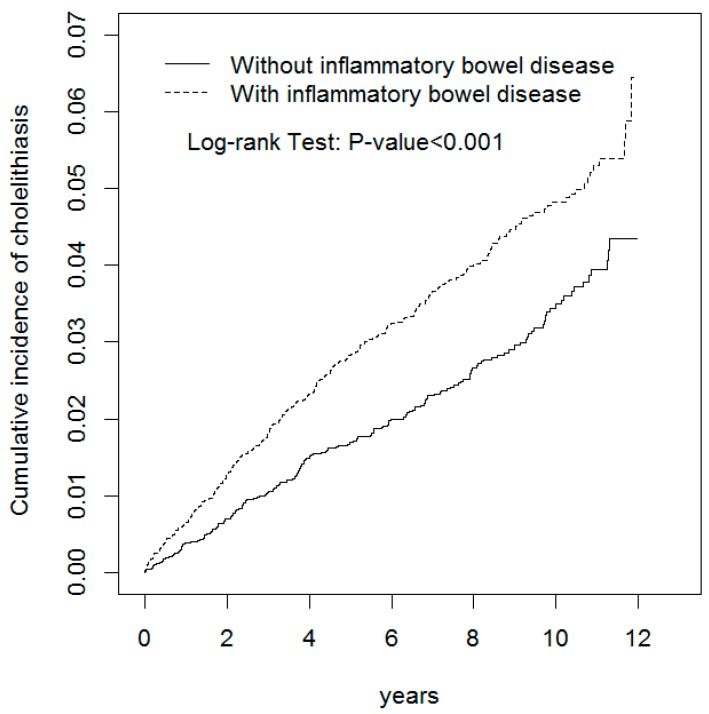
Cumulative incidences of cholelithiasis among patients with (dashed line) and without (solid line) inflammatory bowel disease.

The mean follow-up times in the IBD and non-IBD cohorts were 7.15 years (standard deviation (SD) = 3.22) and 7.14 years (SD = 3.23; data not shown). Figure 1 indicates that at the end of follow-up, the cumulative incidence of cholelithiasis was 2.07% higher in the IBD cohort than in the non-IBD cohort (*p* < 0.001). As the follow-up duration increased, the risk of developing cholelithiasis in the IBD cohort increased compared with that in the non-IBD cohort.

Table 2 presents the incidence of cholelithiasis and its risk factors. Overall, the IBD cohort had a higher cholelithiasis incidence (5.21 per 1000 person-years) than did the non-IBD cohort (3.49 per 1000 person-years), with a crude HR of 1.49 (95% CI = 1.25–1.78) and an adjusted HR (aHR) of 1.51 (95% CI = 1.27–1.81; Table 2). Compared with patients aged ≤49 years, the risk of developing cholelithiasis was 1.99-fold higher in those aged 50–64 years (95% CI = 1.57–2.52) and was 2.80-fold higher in those aged ≥65 years (95% CI = 2.15–3.66). The risk of developing cholelithiasis was higher in women than in men. Moreover, the risk of developing cholelithiasis was higher in patients with hypertension (aHR = 1.28, 95% CI = 1.01–1.61) or COPD (aHR = 1.43, 95% CI = 1.13–1.81).

Table 3 lists the incidence of cholelithiasis stratified by age, sex, and comorbidities and presents the HRs measured using the Cox model for patients with and without IBD. The age-specific aHRs of cholelithiasis across the IBD to non-IBD cohorts were significant for patients aged ≤49 years (aHR = 1.47, 95% CI = 1.08–2.00) and those aged 50–64 years (aHR = 1.80, 95% CI = 1.30–2.50). The relative risk of developing cholelithiasis in the IBD cohort was significantly higher than that in the non-IBD cohort for both women (aHR = 1.46, 95% CI = 1.16–1.84) and men (aHR = 1.60, 95% CI = 1.22–2.11). The relative risk of developing cholelithiasis in the IBD cohort was significantly higher than that in the non-IBD cohort for patients without comorbidities (aHR = 1.73, 95% CI = 1.28–2.36) and those with comorbidities (aHR = 1.39, 95% CI = 1.12–1.72).

Table 4 presents the relative overall risks of cholelithiases, including GSD and non-GSD-associated cholelithiasis, in patients with CD or UC. Patients with CD were 1.87-fold more likely to develop cholelithiasis than patients without IBD (95% CI = 1.34–2.61). Compared with patients without IBD, the risk of developing cholelithiasis was also higher in patients with UC (aHR = 1.47, 95% CI = 1.22–1.76). Compared with patients without IBD, patients with CD exhibited a 1.76-fold increased risk of developing GSD (95% CI = 1.23–2.53), and those with UC exhibited a 1.44-fold increased risk of developing GSD (95% CI = 1.19–1.75). Compared with patients without IBD, patients with CD exhibited a 2.78-fold increased risk of developing non–GSD-associated cholelithiasis (95% CI = 1.18–6.51). However, patients with UC did not exhibit a higher risk of developing non-GSD-associated cholelithiasis.

## 4. Discussion

Consistent with studies conducted in Western countries, most of the patients with IBD in our study were aged <49 years and female [19]. However, the ratio of UC to CD was approximately 8.39 in our study, which is closer to the results of studies conducted in the Asia-Pacific region [20]. The reported epidemiology of IBD in the Chinese population, including the incidence, gender predominance, and the ratio of UC to CD, varies greatly [20,21]. In addition to ethnicity, the environment and lifestyle also affect the epidemiology of IBD. Most studies were conducted before 2007, when disease awareness and diagnostic tools were poor. We acknowledge that more studies are required to clarify the epidemiology of IBD in the Asia-Pacific region.

The association between IBD and cholelithiasis demonstrated in this study was unlikely observed by chance, because the multivariable Cox proportional hazards regression analysis employed to assess the association between IBD and cholelithiasis was adjusted for age, sex, and comorbidities of hyperlipidemia, diabetes, liver cirrhosis, hypertension, COPD, stroke, CAD, and HCV infection. Furthermore, the results consistently indicated that the contribution of IBD to the development of cholelithiasis was greater among middle-aged patients, men, and patients without comorbidities (Table 2), even though the risk of developing cholelithiasis was greater among older adults, women, and patients with comorbidities (Table 1). In addition, compared with the non-IBD cohort, the risk of developing cholelithiasis in the IBD cohort increased with the follow-up duration (Figure 1). All these findings suggest that IBD is independently associated with an increased risk of developing cholelithiasis. However, the two sets of data may be related but not necessarily causally related and the aHRs are significant but they are relatively low (Table 2). Therefore, more studies are needed to clarify the causal relationship between IBD and cholelithiasis.

Notably, age, rather than IBD, was the key risk factor for cholelithiasis, and the contribution of IBD to the development of cholelithiasis was nonsignificant for older adults in our study (Table 2). Previous studies have consistently identified a close association between CD and the development of cholelithiasis; however, the relationship between UC and the development of cholelithiasis remains controversial [6]. In the current large population-based cohort study, CD was found to be closely related to the development of cholelithiases, including GSD alone and cholelithiasis without GSD. However, UC was only related to the development of GSD alone. Notably, the contribution of CD to the development of GSD alone was more critical than that of UC, because the aHR for CD was higher than that for UC in our study (Table 4).

Although more laboratory studies are required to ascertain the mechanisms underlying the association between IBD and the development of cholelithiasis, the pathophysiology of the contribution of IBD to the development of cholelithiasis may be as follows. First, bile acid malabsorption and depletion in the ileum due to impaired bile enterohepatic circulation caused by inflammation or ileostomy may predispose individuals to the development of cholesterol GSD [7]. Second, the unabsorbed bile acids solubilize bilirubin in the colon, increasing the risk of pigment stone formation in the gallbladder [8]. Third, dysbiosis in patients with IBD may lead to bile acid dysmetabolism, thus increasing the risk of developing cholelithiasis [22]. Fourth, reduced gallbladder motility in patients with CD has been observed to enhance the development of GSD [9]. Fifth, activation of the Th1-mediated proinflammatory immune response is mainly found among patients with CD, and this can enhance the development of cholesterol GSD [23]. However, the activation of the Th2-mediated immune response, rather than the Th1-mediated immune response, has been found in patients with UC, and this is not related to the development of cholesterol GSD. Sixth, primary sclerosing cholangitis, which is mainly found in patients with UC, can lead to stone formation due to chronic inflammation and fibrosis in the intrahepatic and extrahepatic biliary trees [11]. Finally, hemolysis induced by IBD-related drugs can increase the risk of pigment stone formation in patients with GSD [10].

Our study had several merits. This is the largest population-based study to demonstrate the association between IBD and the risk of developing cholelithiasis in the Asia-Pacific region. In addition, this study is the first to indicate that CD is closely related to the development of GSD alone and non-GSD cholelithiasis. Moreover, our study is the first to demonstrate that UC is related to the development of GSD alone but not to non-GSD cholelithiasis [6,10]. Our longitudinal, rather than cross-sectional, approach provided a suitable opportunity to evaluate the temporal association between IBD and cholelithiasis. Furthermore, the NHI program provides a reliable patient sample in Taiwan because this program covers approximately 99% of Taiwan’s residents.

Our study also had some limitations. First, some confounding factors may have been overlooked in this study due to the inherent unavailability of data regarding lifestyle, socioeconomic and educational status, genetic history, and definite body weight and height in the LHID2000, all of which can affect the development of IBD and cholelithiasis. For adjustment, we replaced body mass index with obesity and smoking habits with COPD diagnosis. Obesity was coded when the clinically recorded body mass index was >30 kg/m^2^; the significance of obesity was minimal in our study because of its low prevalence in the IBD cohort. However, we acknowledge that the factors of age, sex, and comorbidities of hyperlipidemia, diabetes, liver cirrhosis, hypertension, COPD, stroke, CAD, and hepatitis C virus infection might be over-adjusted since hyperlipidemia, diabetes, liver cirrhosis, hypertension, COPD, stroke, and CAD could be other effects of the initial liver disease. Furthermore, IBD could be the consequence of initial liver disease and would affect the aHRs of IBD in contributing to the development of cholelithiasis. Second, although the relationships of the severity, extension, and management of IBD with the development of cholelithiasis have been controversial in previous studies, we could not assess these associations because the data were unavailable in the LHID2000 [24,25]. Third, we acknowledge the inherent limitation of an observational study to clarify the pathogenesis and the causal relationship of the association between IBD and cholelithiasis. This observational study could not clarify the precise mechanism for the development of cholelithiasis in patients with CD and UC, and the aforementioned potential mechanisms feature bidirectional interactions. It is possible that the manifestations of liver disease of IBD started early and remained asymptomatic, and then over time the initial liver disease could lead to the development of cholelithiasis before the awareness of IBD. Therefore, additional laboratory studies are required to ascertain the potential mechanisms of IBD for the development of cholelithiasis.

## 5. Conclusions

Our population-based cohort study demonstrated a close relationship between CD and cholelithiases, including GSD alone and non-GSD-associated cholelithiasis. However, UC is only related to the development of GSD alone. Because IBD is a chronic disease, clinicians should survey the presence of cholelithiasis in patients with IBD when monitoring extraintestinal manifestations. Furthermore, more studies are required to ascertain the actual mechanisms of IBD that result in a predisposition to the development of cholelithiasis.

## Figures and Tables

**Table 1:** Demographic characteristics and comorbidities in the cohorts with and without inflammatory bowel disease.

Variable	Inflammatory Bowel Disease		*p*-Value
	No	Yes	
	N = 8186	N = 8186	
Age, year			0.99
≤49	4691 (57.3)	4820 (58.9)	
50–64	1868 (22.8)	1848 (22.6)	
65+	1627 (19.9)	1518 (18.5)	
Mean ± SD ^†^	47.9 (17.2)	47.7 (16.8)	0.32
Sex			0.77
Female	4323 (52.8)	4304 (52.6)	
Male	3863 (47.2)	3882 (47.4)	
Comorbidity			
Hyperlipidemia	1457 (17.8)	1451 (17.7)	0.90
Diabetes	559 (6.83)	563 (6.88)	0.90
Liver cirrhosis	95 (1.16)	81 (0.99)	0.29
Alcohol-related illness	257 (3.14)	265 (3.24)	0.72
Hypertension	2324 (28.4)	2283 (27.9)	0.48
COPD	912 (11.1)	924 (11.3)	0.77
Obesity	96 (1.17)	95 (1.16)	0.94
Stroke	231 (2.82)	227 (2.77)	0.85
CAD	1291 (15.8)	1260 (15.4)	0.50
Hepatitis B virus	247 (3.02)	243 (2.97)	0.85
Hepatitis C virus	101 (1.23)	109 (1.33)	0.58

Chi-squared test; ^†^: *t*-test; COPD = chronic obstructive pulmonary disease; CAD = coronary artery disease.

**Table 2:** Incidence of cholelithiasis and its risk factors.

Variable	Event	PY	Rate ^#^	Crude HR ^&^ (95% CI)	Adjusted HR ^†^ (95% CI)
Inflammatory bowel disease					
No	204	58,413	3.49	1.00	1.00
Yes	305	58,535	5.21	1.49 (1.25, 1.78) ***	1.51 (1.27, 1.81) ***
Age, year					
≤49	169	71,822	2.35	1.00	1.00
50–64	155	26,558	5.84	2.47 (1.99, 3.08) ***	1.99 (1.57, 2.52) ***
65+	185	18,568	9.96	4.21 (3.42, 5.19) ***	2.80 (2.15, 3.66) ***
Sex					
Female	295	62,540	4.72	1.20 (1.01, 1.43) *	1.21 (1.01, 1.44) *
Male	214	54,408	3.93	1.00	1.00
Comorbidity					
Hyperlipidemia					
No	362	98,034	3.69	1.00	1.00
Yes	147	18,913	7.77	2.09 (1.73, 2.54) ***	1.13 (0.91, 1.40)
Diabetes					
No	443	110,523	4.01	1.00	1.00
Yes	66	6424	10.3	2.54 (1.96, 3.29) ***	1.29 (0.97, 1.70)
Liver cirrhosis					
No	499	116,153	4.30	1.00	1.00
Yes	10	795	12.6	2.87 (1.53, 5.37) **	1.53 (0.79, 2.96)
Alcohol-related illness					
No	491	114,124	4.30	1.00	1.00
Yes	18	2824	6.37	1.45 (0.91, 2.33)	-
Hypertension					
No	266	87,340	3.05	1.00	1.00
Yes	243	29,608	8.21	2.68 (2.25, 3.19) ***	1.28 (1.01, 1.61) *
COPD					
No	403	106,079	3.80	1.00	1.00
Yes	106	10,869	9.75	2.68 (2.25, 3.19) ***	1.43 (1.13, 1.81) **
Obesity					
No	504	115,912	4.35	1.00	1.00
Yes	5	1036	4.83	1.09 (0.45, 2.63)	-
Stroke					
No	484	114,699	4.22	1.00	1.00
Yes	25	2248	11.1	2.58 (1.72, 3.86) ***	1.13 (0.75, 1.72)
CAD					
No	363	101,005	3.59	1.00	1.00
Yes	146	15943	9.16	2.54 (2.09, 3.07) ***	1.13 (0.90, 1.42)
HBV					
No	496	114,123	4.35	1.00	1.00
Yes	13	2825	4.60	1.05 (0.60, 1.82)	-
HCV					
No	497	115,813	4.29	1.00	1.00
Yes	12	1135	10.6	2.44 (1.38, 4.33) **	1.54 (0.85, 2.82)

PY: person-years; Rate ^#^: incidence rate per 1000 person-years; Crude HR ^&^: relative hazard ratio; Adjusted HR ^†^: multivariable analysis including age, sex, and comorbidities of hyperlipidemia, diabetes, liver cirrhosis, hypertension, COPD, stroke, CAD, and HCV; * *p* < 0.05, ** *p* < 0.01, *** *p* < 0.001.

**Table 3:** Cox model-measured incidence of cholelithiasis stratified by age, sex, and comorbidities and hazard ratios for patients with and without inflammatory bowel disease.

Variables	Inflammatory Bowel Disease						Crude HR ^&^ (95% CI)	Adjusted HR ^†^ (95% CI)
	No			Yes				
	Event	PY	Rate ^#^	Event	PY	Rate ^#^		
Age, years								
≤49	66	35,488	1.86	103	36,335	2.83	1.52 (1.12, 2.07) **	1.47 (1.08, 2.00) *
50–64	56	13,365	4.19	99	13,193	7.50	1.79 (1.29, 2.48) ***	1.80 (1.30, 2.50) ***
65+	82	9561	8.58	103	9008	11.4	1.33 (1.00, 1.78)	1.33 (1.00, 1.78)
Sex								
Female	120	31,232	3.84	175	31,308	5.59	1.45 (1.15, 1.83) **	1.46 (1.16, 1.84) **
Male	84	27,181	3.09	130	27,227	4.77	1.54 (1.17, 2.03) **	1.60 (1.22, 2.11) ***
Comorbidity								
No	65	36,320	1.79	108	34,726	3.11	1.73 (1.27, 2.36) ***	1.73 (1.28, 2.36) ***
Yes	139	22,092	6.29	197	23,808	8.27	1.32 (1.06, 1.64) *	1.39 (1.12, 1.72) **

PY: person-years; Rate ^#^: incidence rate per 1000 person-years; Crude HR ^&^: relative hazard ratio; Adjusted HR ^†^: multivariable analysis including age, sex, and comorbidities of hyperlipidemia, diabetes, liver cirrhosis, hypertension, COPD, stroke, CAD, and HCV; * *p* < 0.05, ** *p* < 0.01, *** *p* < 0.001.

**Table 4:** Cox model-measured incidence of cholelithiasis and hazard ratios for patients with and without inflammatory bowel disease.

Variables (ICD-9 Code)	Event	Rate ^#^	Crude HR ^&^ (95% CI)	Adjusted HR ^†^ (95% CI)
Cholelithiasis				
Non-IBD cohort (N = 8186)	204	3.49	1 (Reference)	1 (Reference)
IBD				
Crohn’s disease (N = 872)	42	7.13	2.03 (1.46, 2.83) ***	1.87 (1.34, 2.61) ***
Ulcerative colitis (N = 7314)	263	5.00	1.43 (1.19, 1.72) ***	1.47 (1.22, 1.76) ***
Gallbladder stones				
Non-IBD cohort (N = 8186)	182	3.12	1 (Reference)	1 (Reference)
IBD				
Crohn’s disease (N = 872)	35	5.94	1.90 (1.32, 2.73) ***	1.76 (1.23, 2.53) **
Ulcerative colitis (N = 7314)	230	4.37	1.40 (1.15, 1.70) ***	1.44 (1.19, 1.75) ***
Non-gallbladder stones				
Non-IBD cohort (N = 8186)	22	0.38	1 (Reference)	1 (Reference)
IBD				
Crohn’s disease (N = 872)	7	1.19	3.14 (1.34, 7.35) **	2.78 (1.18, 6.51) *
Ulcerative colitis (N = 7314)	33	0.63	1.66 (0.97, 2.84)	1.70 (0.99, 2.91)

Rate ^#^: incidence rate per 1000 person-years; Crude HR ^&^: relative hazard ratio; Adjusted HR ^†^: multivariable analysis including age, sex, and comorbidities of hyperlipidemia, diabetes, liver cirrhosis, hypertension, COPD, stroke, CAD, and HCV; * *p* < 0.05, ** *p* < 0.01, *** *p* < 0.001.

## Abbreviations

IBD	inflammatory bowel disease
CD	Crohn’s disease
UC	ulcerative colitis
aHR	adjusted hazard ratio
CI	confidence interval
LHID2000	Longitudinal Health Insurance Research Database 2000
GSD	gallbladder stone disease
CBD	common bile duct
IHSs	intrahepatic stones
NHI	National Health Insurance
ICD-9-CM	International Classification of Diseases, Ninth Revision, Clinical Modification
COPD	chronic obstructive pulmonary disease
CAD	coronary artery disease
HRs	hazard ratios
SDs	standard deviations
NHIRD	National Health Insurance Research Database
